# Guidelines for the Initial Assessment of Respiratory Distress in the Emergency Department

**DOI:** 10.1016/j.aicoj.2025.100005

**Published:** 2026-01-20

**Authors:** P. Le Borgne, A.W. Thille, J. Guenezan, N. Aissaoui, A.-S. Boureau, C. Bally, F. Balen, A. Basset, P. Bilbault, F. Boissier, Y.-E. Claessens, M. Decavèle, J.-L. Diehl, D. Douillet, A. Guillon, P. Hausfater, F. Javaudin, M. Jezequel, K. Kuteifan, E. L’Her, N. Marjanovic, E. Maury, M. Ohana, C. Pichereau, P. Ray, P.-G. Reuter, N. Tiberti, G. Voiriot, Y. Yordanov, P. Le Conte, N. Terzi

**Affiliations:** a Université de Strasbourg, Service des Urgences, Hôpitaux Universitaires de Strasbourg, F-67000 Strasbourg, France; b Université de Poitiers, Service de Médecine Intensive Réanimation, CHU de Poitiers, F- 86000 Poitiers, France; c Université de Poitiers, Service des Urgences, SAMU, SMUR, CHU de Poitiers; Faculté de Médecine et de Pharmacie, F- 86000 Poitiers, France; d Université Paris Cité, Service de Cardiologie, Hôpitaux Européens Georges Pompidou, AP-HP, F-75010 Paris, France; e Université de Nantes, Pole de Gérontologie Clinique, CHU Nantes, F-44000 Nantes, France; f Service de Réanimation polyvalente/USC/Déchocage, Centre Hospitalier Annecy Genevois, F74000 Annecy, France; g Université Toulouse III, Pôle de Médecine d'Urgence, CHU Toulouse, F-31000 Toulouse, France; h Département de Médecine d'Urgence, CHRU Brest, F-29200 Brest, France; i Département de Médecine d'Urgence, Centre Hospitalier Princesse Grace, MC-98002, Monaco; j Sorbonne Université, Service de Médecine Intensive - Réanimation (Département R3S), Site Pitié-Salpêtrière, AP-HP, F-75010 Paris, France; k Université Paris Cité, Service de Médecine Intensive Réanimation, Hôpitaux Européens Georges Pompidou, AP-HP, F-75010 Paris, France; l Université d’Angers, Département de Médecine d’Urgence, CHU Angers, F-49100, Angers, France; m Université de Tours, Service de Médecine Intensive Réanimation, CHU Tours, F-37000, Tours, France; n Sorbonne Université, Service des Urgences, APHP-Sorbonne Université, Site Pitié-Salpêtrière, AP-HP, F-75010 Paris, France; o Université de Nantes, Service des Urgences, CHU de Nantes, F-44000 Nantes, France; p Service de Réanimation Médicale, Centre Hospitalier de St Brieuc, F-22000 St Brieuc, France; q Service de Réanimation Médicale, GHRMSA, Hôpital Emile Muller, F-68100 Mulhouse, France; r Université de Brest, Service de Médecine Intensive et de Réanimation, CHRU de Brest, F-29200 Brest, France; s Sorbonne Université, Service de Médecine Intensive et de Réanimation, Hôpital Saint-Antoine, AP-HP, F-75571 Paris, France; t Service de Radiologie, Nouvel Hôpital Civil, Hôpitaux Universitaires de Strasbourg, F-67000 Strasbourg, France; u Service de Médecine Intensive Réanimation, Centre Hospitalier Intercommunal de Poissy Saint Germain, F-78300, Poissy, France; v Département Universitaire de Médecine d’Urgence, Université Bourgogne Europe, CHU de Dijon, Dijon, France; w Université de Rennes, Service SAMU 35/SMUR/Urgences Adultes, CHU Rennes, F-35000 Rennes, France; x Aix-Marseille Université, Structure des Urgences, Centre Hospitalier Intercommunal Aix-Pertuis, F-13616 Aix-en-Provence, France; y Sorbonne Université, Service de Médecine Intensive Réanimation, Hôpital Tenon, AP-HP, F-75020 Paris, France; z Université de Lyon, Service des Urgences-SAMU 69, Hôpital Edouard Herriot, HCL, F-69001 Lyon, France; aa Université de Rennes, Service de Médecine Intensive - Réanimation, CHU de Rennes, F-35033, Rennes, France

**Keywords:** Respiratory distress, Dyspnea, Triage, Acute respiratory failure, Emergency department, Severity assessment

## Abstract

**Objective:**

The French Society of Emergency Medicine (SFMU) and the French Intensive Care Society (SRLF) present formalized expert recommendations from a multidisciplinary panel on the initial assessment of respiratory distress in adult patients presenting to the Emergency Department.

**Design:**

A group of 30 French experts from the SFMU and FICS was assembled. Any potential conflicts of interest were officially declared at the start of the guidelines development process, which was conducted independently of any industry funding. The authors followed the GRADE (Grading of Recommendations Assessment, Development, and Evaluation) methodology to assess the level of evidence from the literature.

**Methods:**

The aim of this expert panel was to define evidence-based recommendations for the initial assessment of respiratory distress in the emergency setting. Three key areas were defined: (1) assessment of severity in respiratory distress; (2) initial assessment and triage in respiratory distress; (3) diagnostic approach for respiratory distress. For each area, the goal of the recommendations was to address a set of questions formulated by the experts following the PICO model (“Population, Intervention, Comparison, Outcome”). Based on these questions, a comprehensive literature search was conducted for the last 20 years using predefined keywords according to PRISMA guidelines. The quality of the data was analyzed using the GRADE method. The recommendations were formulated using the GRADE methodology, and then voted on by all experts using the GRADE Grid method.

**Results:**

The expert consensus process, based on the GRADE methodology, resulted in 13 clinical questions yielding 20 recommendations. For three of these questions, however, no recommendation could be issued due to insufficient evidence. After two rounds of voting and several amendments, a strong consensus was reached on the recommendations. Among these, two were supported by high-quality evidence and resulted in a strong recommendation (GRADE 1); six were based on moderate-quality evidence and led to a conditional recommendation (GRADE 2); and twelve were based on expert opinion, reflecting a low level of evidence. Finally, for 3 questions, no recommendation could be formulated.

**Conclusion:**

A strong consensus was reached among the experts on 20 of the recommendations. This work provides updated recommendations for the initial assessment of respiratory distress in adult patients presenting to the Emergency Department.

## Introduction

These Clinical Practice Guidelines (CPGs) are the result of a joint expert consensus process initiated by the French Society for Emergency Medicine (SFMU) and the French Intensive Care Society (FICS). They aim to address the clinical issue of respiratory distress in prehospital and in-hospital emergency settings. The recommendations are structured around clearly defined PICO (Patient, Intervention, Comparison, Outcome) questions, covering the assessment of severity (from prehospital dispatch to Emergency Department triage), diagnostic approach (clinical scores, biomarkers, imaging), therapeutic decision-making, monitoring, and patient disposition. However, before addressing these clinical questions, this guideline emphasizes the importance of defining key terms and clarifying the current conceptual framework for dyspnea**, respiratory distress,** and acute respiratory failure.

**Dyspnea** (subjective symptom):

Dyspnea refers to the subjective sensation of “not breathing normally,” “shortness of breath,” or “suffocating.” Although less studied and recognized than pain, dyspnea shares many epidemiological, clinical, neurophysiological, and psychological features with it. Both symptoms are frequent, multidimensional biopsychosocial experiences with sensory, emotional, cognitive, behavioral, cultural, and spiritual components [1,2]. Unlike pain, dyspnea often includes a distinct sense of dread. It signals a critical threat to homeostasis and is strongly associated with negative emotions such as anxiety and fear of death [3]. For dyspneic patients, breathing itself becomes a source of suffering, threat, and constant reminder of imminent death: “It feels like fear—you don’t think you’ll ever catch your breath again. It brings panic, fear, and a sense of tightness;” “When breathlessness peaked, I truly thought I was going to die” [4].

An international expert consensus, under the auspices of the European Respiratory Society (ERS) and the European Society of Intensive Care Medicine (ESICM), recently proposed an updated, operational definition of dyspnea that reflects its negative experiential intensity: “Dyspnea is the unpleasant and distressing perception of one’s breathing” [5]. It is important to distinguish dyspnea from breathlessness, which refers to a normal physiological sensation experienced during exertion in healthy individuals. Breathlessness is not necessarily unpleasant, does not evoke fear of suffocation, and can be alleviated by reducing physical effort [6].

From an epidemiological standpoint, dyspnea is one of the most frequent symptoms encountered in clinical medicine - either acutely (e.g., asthma exacerbation, pneumothorax, pulmonary embolism, pneumonia, acute heart failure) or chronically (e.g., COPD, chronic heart failure, cancer, neuromuscular disorders, obesity, deconditioning, or psychological disorders) [[6], [7], [8]]. Chronic dyspnea affects an estimated 20% of the global population. In emergency settings, respiratory distress accounts for 5–10 % of all ED presentations, approximately 10% of hospital admissions, and nearly 20% of ICU admissions [[9], [10], [11], [12]]. Importantly, observable signs of respiratory distress may be disconnected from the subjective experience of dyspnea, and are thus not reliable proxies for the patient’s internal respiratory burden [13]. According to ERS and ESICM, dyspnea can only be perceived and reported by the patient experiencing it [14]. For these reasons, this guideline does not focus on the subjective dimension of dyspnea (e.g., intensity, emotional or neurovegetative impact), but rather uses the term respiratory distress as a pragmatic label encompassing the observable clinical manifestations of ventilatory compromise. Most studies cited in this document do not report the prevalence or intensity of dyspnea per se. While dyspnea is the primary presenting symptom in the ED, clinical severity is primarily assessed based on objective signs of respiratory distress, such as respiratory rate, use of accessory muscles, or hypoxemia.

**Respiratory distress** (clinical observation)

Respiratory distress is defined as the constellation of observable clinical signs indicating a significant impairment of respiratory function. These signs may be associated with hemodynamic or neurological compromise and reflect a mismatch between the ventilatory demand and the capacity of the respiratory system to meet it. The clinical picture typically involves manifestations related to impaired gas exchange, such as cyanosis; signs of increased respiratory effort, including tachypnea, excessive use of accessory inspiratory muscles (notably supraclavicular, sternal, subxiphoid, or intercostal retractions), nasal flaring, abdominal paradox, or Campbell’s sign; and signs of decompensation or exhaustion of the respiratory system, such as the inability to speak in full sentences, hypercapnic coma, cognitive slowing, profuse sweating, thoraco-abdominal asynchrony, bradypnea, and eventually respiratory arrest. It is worth noting that upper airway obstruction (for instance, laryngeal obstruction) may cause prominent audible signs of respiratory distress upon inspection, without necessarily inducing hypoxemia or hypercapnia.

Acute respiratory failure (biological definition)

Acute respiratory failure corresponds to the inability of the respiratory system to ensure adequate gas exchange, and is defined by objective biological criteria. It is characterized by an acute impairment of hematosis, as evidenced by arterial blood gas analysis. This includes hypoxemia, defined as a partial pressure of oxygen (PaO₂) below 60 mmHg or a peripheral oxygen saturation (SpO₂) below 90% in ambient air, or a PaO₂/FiO₂ ratio below 300 mmHg in patients receiving supplemental oxygen. In addition, hypercapnia - defined by a partial pressure of carbon dioxide (PaCO₂) above 45 mmHg -may be present, with or without associated acidemia. Type I acute respiratory failure refers to isolated hypoxemia, whereas type II involves hypercapnia and often acid-base disturbances. This definition is strictly biological and does not take into account the presence of clinical respiratory distress or the patient’s subjective experience of dyspnea. Notably, it may also encompass hypoxemic conditions related to anatomical right-to-left shunting, such as patent foramen ovale, cyanotic congenital heart disease, pulmonary hypertension, or pulmonary arteriovenous malformations, even in the absence of intrinsic parenchymal lung disease.

Before addressing the modalities of clinical assessment, it is helpful to briefly recall the main etiologies of respiratory distress in emergency settings, along with key elements of initial triage. The causes of respiratory distress can be broadly classified into two main categories: on the one hand, cardiopulmonary etiologies - representing the vast majority of cases encountered in the ED - such as acute heart failure, pneumonia, pulmonary embolism, acute exacerbations of COPD or asthma, and pneumothorax; on the other hand, less frequent causes, including metabolic disorders (such as acidosis or severe anemia), neuromuscular dysfunction, and psychogenic dyspnea. Initial triage should be based on a rapid assessment integrating respiratory rate, oxygen saturation (SpO₂), clinical signs of respiratory distress, and the patient's relevant medical history, including prior comorbidities. In this guideline, we choose to focus exclusively on cardiopulmonary causes of respiratory distress.

Although dyspnea is a subjective symptom, its quantification plays a central role not only in clinical assessment, therapy, and monitoring, but also in clinical research. Validated tools such as the VAS, Borg, or mMRC scales may be used, especially in chronic respiratory patients; however, their routine use in EDs is inconsistent and falls outside the scope of these guidelines.

Finally, in 2024, the European Society of Intensive Care Medicine (ESICM) published an international statement emphasizing the importance of assessing and grading dyspnea in patients receiving mechanical ventilation in the intensive care unit (ICU) setting [5]. This expert consensus focused primarily on intubated or non-invasively ventilated patients, addressing the neurophysiological, psychological, and prognostic implications of breathlessness in critically ill individuals. In contrast, the present guidelines specifically address the evaluation and early management of respiratory distress as a presenting symptom in spontaneously breathing patients admitted to the Emergency Department (ED). Our recommendations are thus distinct in scope and methodology, focusing on the triage, diagnostic work-up, and initial treatment of patients presenting with respiratory distress in the ED, prior to any escalation to mechanical support. While symptom severity remains clinically relevant, its quantification plays a secondary role in the emergency setting, where the priority lies in recognizing signs of respiratory distress and initiating timely, syndrome-oriented management. Nevertheless, the two approaches may be viewed as complementary. Standardized dyspnea assessment tools - although rarely implemented in the ED - could help stratify patients earlier, improve communication between prehospital, ED and ICU teams, and provide continuity in patient monitoring. Future research should explore how symptom-based severity grading might enhance prognostic accuracy and support clinical decision-making in spontaneously breathing patients.

## Methods

These guidelines are the result of a collaborative effort led by a panel of 30 experts in emergency medicine and intensive care medicine, convened by the French Society for Emergency Medicine (SFMU) and the French Intensive Care Society (FICS), at the request of their respective Executive Committees. All experts completed a conflict-of-interest disclosure form prior to initiating their work.

After defining the objectives and the methodological framework of the project, the organizing committee determined the scope of these Clinical Practice Guidelines and formulated the questions to be addressed. Each question was assigned to several experts for review and drafting. All questions followed the PICO format (Population, Intervention, Comparison, Outcome). A systematic literature review was conducted using the PubMed™, Cochrane™, and www.clinicaltrials.gov databases. Eligible publications were written in English or French. Priority was given to recent evidence, with an appraisal hierarchy ranging from meta-analyses and randomized controlled trials (RCTs) to observational studies. The sample size and relevance of each study were assessed individually. For each reference, the level of evidence was determined based on study type and methodological quality. Recommendations were formulated using the terminology and grading system established by the SFMU and the FICS. High-level evidence led to strong recommendations (GRADE 1+ or 1−: "it is recommended to…" or "it is not recommended to…"), whereas moderate or low-level evidence supported conditional recommendations (GRADE 2+ or 2−: "it is probably recommended to…" or "it is probably not recommended to…"). In the absence of sufficient evidence, expert opinions were provided using the phrasing: “the experts suggest…”.

All recommendation statements were presented and discussed individually. The objective was not to enforce full consensus but to identify areas of agreement, divergence, or uncertainty. Each recommendation was then independently rated by all panel members using a 1-to-9 Likert scale, where 1–3 represented disagreement, 4–6 uncertainty, and 7–9 agreement. Consensus was assessed using the GRADE Grid method. A strong agreement was defined as at least 70% of experts rating the recommendation in the agreement zone (7–9) and fewer than 20% in the disagreement zone (1–3). In the absence of strong consensus, recommendations were reformulated and re-evaluated in subsequent voting rounds to achieve agreement. Weak consensus was defined as agreement by 50–70% of experts and fewer than 20% in disagreement. Expert opinions - by definition ungraded due to insufficient supporting literature - were required to meet the criteria for strong agreement (≥70% agreement) to be retained.

## Results

These guidelines were structured around three main areas of application: (1) the assessment of severity in the context of respiratory distress; (2) the initial evaluation and triage of patients presenting with respiratory distress; and (3) the diagnostic workup of respiratory distress in emergency medical settings (both prehospital and in-hospital).

The expert synthesis and application of the GRADE methodology led to the formulation of 20 recommendations and 3 instances where no recommendation could be issued, based on a total of 13 clinical questions. Following two rounds of voting and a limited number of amendments, strong agreement was reached for the majority of recommendations and the proposed algorithm. Among the 20 recommendations, 2 were supported by high-quality evidence (GRADE 1), 6 by moderate quality evidence (GRADE 2), and 12 were based on expert opinion. For 3 questions, the panel was unable to formulate a recommendation due to insufficient or inconclusive evidence.

### Field 1 - assessment of severity

In patients presenting with respiratory distress during medical dispatch triage, what clinical criteria should prompt the dispatch of a physician-staffed advanced life support (ALS) unit rather than a non-medicalized rescue team?

**Table:** 

R1.1.1. The experts suggest that the following clinical severity signs should be actively sought during the emergency call interview, as they may justify dispatching a physician-staffed advanced life support unit: tachypnea (respiratory rate >25 breaths/min), thoraco-abdominal asynchrony, inability to speak full sentences, peripheral cyanosis, profuse sweating, and altered level of consciousness. These signs should be assessed either directly with the patient or via a bystander, and videoconferencing may be used to assist in their identification
Expert opinion (strong agreement)

### Rationale

Emergency calls for respiratory distress are common and represent a major challenge in prehospital triage. In a recent Danish study, dyspnea was the fifth most frequent reason for emergency calls, accounting for 8% of all cases and associated with a 24 -h mortality of 10.5% [15]. To date, no randomized controlled trials have specifically compared a guided versus non-guided strategy for identifying severity criteria in patients with respiratory distress during emergency dispatch. Lindskou et al. prospectively analyzed more than 3, 000 patients with dyspnea managed by ambulance services [16]. Combining a self-reported dyspnea score on a 0-to-10 visual analog scale with vital signs (respiratory rate, oxygen saturation, heart rate, and blood pressure) yielded an area under the curve of 0.72 for predicting ICU admission within 48 h or 30-day mortality. However, such parameters - apart from respiratory rate - are rarely available at the initial dispatch stage. Although a qualitative study involving 12 patients recommended the use of self-assessment tools for dyspnea [17], this approach has not yet been widely adopted in France. Literature specifically addressing telephone triage of dyspnea remains limited. A 2021 meta-analysis by *Ponnapalli* et al. reviewed available modalities for remote assessment of dyspnea and identified five eligible studies [18]. The most commonly evaluated clinical features included respiratory rate, respiratory effort, counting time, and level of consciousness. Counting time was assessed using the Roth score, which is defined by an inability to count to 20 in a single breath or for more than 7 seconds continuously [19]. In a retrospective cohort of 1,425 patients with dyspnea, *Balen* et al. developed a predictive score for identifying patients likely to require ventilatory support, including high-flow oxygen therapy, non-invasive ventilation, or invasive mechanical ventilation [20]. The score incorporated six variables that could potentially be assessed by telephone: long-term treatment with β2-agonists, tachypnea, inability to finish sentences (also assessed via the Roth score), peripheral cyanosis, profuse sweating, and altered consciousness. This score is currently undergoing external validation. Further research is warranted, particularly on the use of videoconferencing during dispatch calls, which has shown promise in pediatric populations [18]. In adults, *Marjanovic et al.* demonstrated that videoconferencing improved the detection of clinical signs of severity in patients with acute dyspnea [21]. During the COVID-19 pandemic, *Larribau* et al. surveyed emergency physicians who used videoconferencing during remote patient assessment [22]; 81.3% reported using it primarily to assess respiratory effort, and 78.5% to evaluate overall clinical status. Notably, physicians reported that videoconferencing influenced their decision-making in 76% of cases.

In patients with respiratory distress managed by a prehospital physician-staffed emergency team, which clinical criteria justify direct admission to the intensive care unit (ICU)?

**Table:** 

R1.2.1. The experts suggest that direct admission to the intensive care unit (ICU) should be proposed for patients receiving invasive mechanical ventilation, and considered for those under non-invasive ventilation, those requiring persistently high oxygen flows, or those presenting with at least one additional organ failure beyond the respiratory system during prehospital management.
Expert opinion (strong agreement)

### Rationale

No studies were identified that specifically compared outcomes in patients with respiratory distress managed by prehospital physician-staffed teams according to whether they were admitted directly to an intensive care unit (ICU) or first assessed in the Emergency Department (ED). However, several studies have demonstrated that delays in ICU admission for patients presenting with respiratory distress are associated with increased mortality, longer durations of mechanical ventilation, and extended ICU length of stay [[23], [24], [25], [26], [27], [28], [29]]. This excess mortality was observed both in patients who received invasive mechanical ventilation in the emergency department and in those who remained on spontaneous ventilation [26,29]. In light of these findings, the expert panel suggests that, in order to reduce delays in critical care admission - and thereby decrease morbidity and mortality - patients requiring ICU-level care should be considered for direct admission from the prehospital setting, or for expedited transfer to the ICU following a brief stabilization period in the ED.

In patients managed in the prehospital setting or admitted to the Emergency Department (ED) with respiratory distress, which physical examination findings are associated with poor outcomes such as death, endotracheal intubation, or ICU admission?

**Table:** 

**R1.3.1.** Patients presenting with tachypnea (respiratory rate >25 breaths per minute) are at increased risk of requiring endotracheal intubation compared to patients without tachypnea. The risks of ICU admission and in-hospital mortality are also higher in this population.
GRADE 2, Moderate level of evidence (strong agreement)
**R1.3.2.** The experts suggest that patients who present with altered consciousness, paradoxical abdominal breathing, or use of accessory inspiratory muscles have an increased risk of in-hospital mortality compared to those without these clinical signs.
Expert opinion (strong agreement)

### Rationale

The analysis of available literature highlights the challenges involved in identifying reliable physical examination findings that predict disease severity in patients presenting with respiratory distress, whether in prehospital or Emergency Department settings. Across studies, the signs and symptoms evaluated are not always consistent, and the definitions of individual clinical variables - such as tachypnea - may vary considerably between publications. Nevertheless, several studies, some involving large cohorts of patients with acute dyspnea (most often in the context of acute exacerbations of chronic obstructive pulmonary disease [COPD]), have identified clinical signs associated with poor outcomes, including the need for endotracheal intubation [[30], [31], [32]] and in-hospital mortality [[33], [34], [35], [36], [37], [38]]. Among these, tachypnea, use of accessory respiratory muscles, and altered mental status have consistently emerged as markers of increased risk [[39], [40], [41]].

### Field 2 - initial assessment, triage and monitoring

In patients admitted to the Emergency Department for respiratory distress, does the use of a structured triage scale reduce morbidity and mortality?

**Table:** 

R2.1.1. The experts suggest that patients presenting with respiratory distress - whether or not they exhibit severity criteria - should be triaged using a structured triage scale upon admission to the Emergency Department, in order to reduce morbidity and mortality.
Expert opinion (strong agreement)

### Rationale

There is currently no high-level evidence directly comparing the impact of structured triage scales versus clinical judgment alone on morbidity and mortality in patients with respiratory distress admitted to Emergency Departments (ED). The first structured triage tool, the Emergency Nursing Classification in Emergency Medicine (Classification Infirmière en Médecine d’Urgence, CIMU), was introduced in 1997. Since then, several triage systems have been implemented internationally, including the Emergency Severity Index (ESI), the Manchester Triage Scale (MTS), the Canadian Triage and Acuity Scale (CTAS), and the French Emergency Nurses Classification in Hospital (FRENCH) scale. In its 2020 national guideline on emergency triage nurses (Infirmier Organisateur de l’accueil, IOA), the French Society for Emergency Medicine (SFMU) recommended the use of a validated, reliable, and reproducible triage scale with four or five levels, tailored to the organization of the national healthcare system [42]. The reliability of the FRENCH scale has been demonstrated in a population of triage nurses (n = 16), with satisfactory inter-rater reproducibility (κ = 0.78; 95%CI [0.71−0.83]) and intra-rater reproducibility (κ = 0.88; 95%CI [0.80−0.94]) [43]. The validity of the FRENCH scale was confirmed in a single-center study involving 26,892 patients by assessing the correlation between triage level and hospitalization rate, with an area under the ROC curve of 0.83 (95%CI [0.82−0.83]). Patients triaged as level 3A had higher hospitalization rates and underwent more diagnostic investigations than those triaged as 3B (p < 0.001). Although this study did not specifically focus on dyspneic patients, dyspnea is a well-identified and common complaint in emergency settings. Based on associated clinical signs, patients presenting with respiratory distress are triaged into categories requiring medical evaluation either immediately (triage level 1), within 20 min (level 2), or within one hour of arrival (level 3) [44]. The MTS uses a five-color code system based on severity: red (immediate), orange (very urgent), yellow (urgent), green (standard), and blue (non-urgent) [45]. In a single-center retrospective study of 4,076 ED patients presenting with dyspnea, *Ausserhofer* et al. demonstrated the safety of the MTS in identifying patients who could safely wait before medical assessment. In this study, 99% of patients triaged to low-priority categories (blue or green) were still alive at 7 days [46]. Regarding the predictive value of the MTS for adverse events, Zaboli et al., in a cohort of 7,055 patients with suspected pulmonary embolism, reported a specificity of 72.6% (95% CI [71.5–73.6]), a sensitivity of 54.2% (95%CI [41.5–66.9]), and a negative predictive value of 99.5% (95%CI [99.4–99.6]) [47]. Collectively, these findings suggest that the use of a validated triage scale by emergency triage nurses (IOA) may help ensure that patients with respiratory distress assigned to low-priority codes can safely wait, while higher-risk patients are prioritized appropriately.

In patients with respiratory distress, does vital signs monitoring reduce morbidity and mortality, and which monitoring modalities should be preferred?

**Table:** 

R2.2.1. The experts suggest that patients presenting with respiratory distress but without severity criteria (triage levels 3A and 3B) should undergo regular monitoring of vital signs, including systolic blood pressure (SBP), heart rate (HR), respiratory rate (RR), body temperature, level of consciousness, and peripheral oxygen saturation (SpO₂), during their stay in the Emergency Department (ED), in order to reduce morbidity and mortality.
Expert opinion (strong agreement)
R2.2.2. The experts suggest that patients presenting with respiratory distress and severity criteria (triage levels 1 and 2) should undergo continuous monitoring of vital signs, including SBP, HR, RR, body temperature, level of consciousness, and SpO₂, throughout their stay in the ED, in order to reduce morbidity and mortality. The experts further encourage that respiratory rate (RR) be measured over a minimum duration of 30 seconds upon arrival and continuously monitored during the entire emergency care period.
Expert opinion (strong agreement)
R2.2.3. The experts suggest using a numerical scale to assess the most suggestive components of dyspnea in patients admitted to the ED, regardless of the presence or absence of severity criteria.
Expert opinion (strong agreement)

### Rationale

There is currently no high-level evidence demonstrating a direct reduction in morbidity and mortality among patients with respiratory distress in the Emergency Department (ED) who undergo continuous vital sign monitoring compared to those who do not. Since 1997, several clinical scoring systems based on physiological parameters - including systolic blood pressure (SBP), heart rate (HR), respiratory rate (RR), body temperature, level of consciousness, peripheral oxygen saturation (SpO₂), and oxygen administration - have been developed to support the monitoring of patients with respiratory distress. A systematic review reported a reduction in mortality and serious adverse events associated with the use of Early Warning Scores (EWS) and staff training, although the heterogeneity of the studies limits the generalizability of the findings [48]. A modified version of EWS, the National Early Warning Score (NEWS2), which incorporates SpO₂ and oxygen therapy, is currently recommended in the United Kingdom for detecting clinical deterioration in patients admitted to emergency settings [49,50]. In a prospective study of 246 patients presenting with respiratory distress, increasing NEWS2 scores were associated with decreased 90-day survival [51]. Regarding the SpO₂/FiO₂ ratio, a retrospective cohort study involving 456 patients hospitalized in a pulmonology unit showed that this ratio could predict ICU transfer, with an area under the ROC curve (AUC) of 0.74 (95% CI: [0.68−0.80]), compared to NEWS2 (AUC = 0.67; 95%CI: [0.61−0.73]) [52]. Although the AUC of the SpO₂/FiO₂ ratio for in-hospital mortality was 0.66 (95%CI: [0.61−0.70]) and higher than any EWS model, the difference was not statistically significant. Based on these observations, *Viglino et al.* developed the Early Warning Score O₂, calculated using the formula: (RR / [SpO₂/FiO₂]) × (HR/100), which has the advantage of being automatically derived from recorded physiological parameters [53]. In a retrospective single-center study including 1,729 patients presenting with dyspnea in the ED, this score showed superior performance, with an AUC of 0.70 (95%CI [0.67−0.74]) compared to the SpO₂/FiO₂ ratio (AUC = 0.70; 95%CI: [0.66−0.73], p = 0.46), NEWS2 (AUC = 0.67; 95%CI: [0.64−0.70], p = 0.02), and NEWS (AUC = 0.66; 95%CI: [0.63−0.70], p < 0.01).

Moreover, a recent multicenter prospective study demonstrated that the modified Sequential Organ Failure Assessment (mSOFA) score outperformed eight other scoring systems in predicting 48 -h in-hospital mortality among patients with respiratory distress in the prehospital setting [54]. As previously discussed, patients with a respiratory rate greater than 25 breaths per minute are at increased risk of endotracheal intubation, ICU admission, and in-hospital mortality, highlighting the crucial role of respiratory rate in monitoring patients with respiratory distress. A multicenter study involving 78 emergency nurses showed significant variability in respiratory rate measurement practices, with only 75.7% using a formal 30-second measurement, compared to 52.6% using brief 12-second assessments (McNemar χ² = 10.32, p = 0.001) [55]. Regarding the subjective assessment of dyspnea, the Modified Borg Scale (MBS) - a numerical scale from 0 (no dyspnea) to 10 (maximum dyspnea) - has been studied in patients with asthma (n = 42) and chronic obstructive pulmonary disease (COPD, n = 60) [56]. In the asthma group, MBS was significantly negatively correlated with changes in peak expiratory flow (PEF) (r = −0.31, p < 0.05) and oxygen saturation (r = −0.26, p < 0.05). In the COPD group, a significant negative correlation was observed between MBS and PEF variation (r = −0.42, p < 0.001), although no correlation was found with oxygen saturation changes. The use of a numerical scale offers a quick and speech-sparing method to assess the patient’s subjective respiratory experience. However, this approach should be validated in larger multicenter studies.

### Field 3 - diagnostic approach

In patients with respiratory distress and suspected pulmonary embolism (PE), how do diagnostic strategies incorporating clinical pre-test probability and scoring systems compare to classical strategies based on age-adjusted D-dimer testing and thoracic CT angiography, in terms of safety and optimization of diagnostic work-up?

**Table:** 

**Fig. 1:**
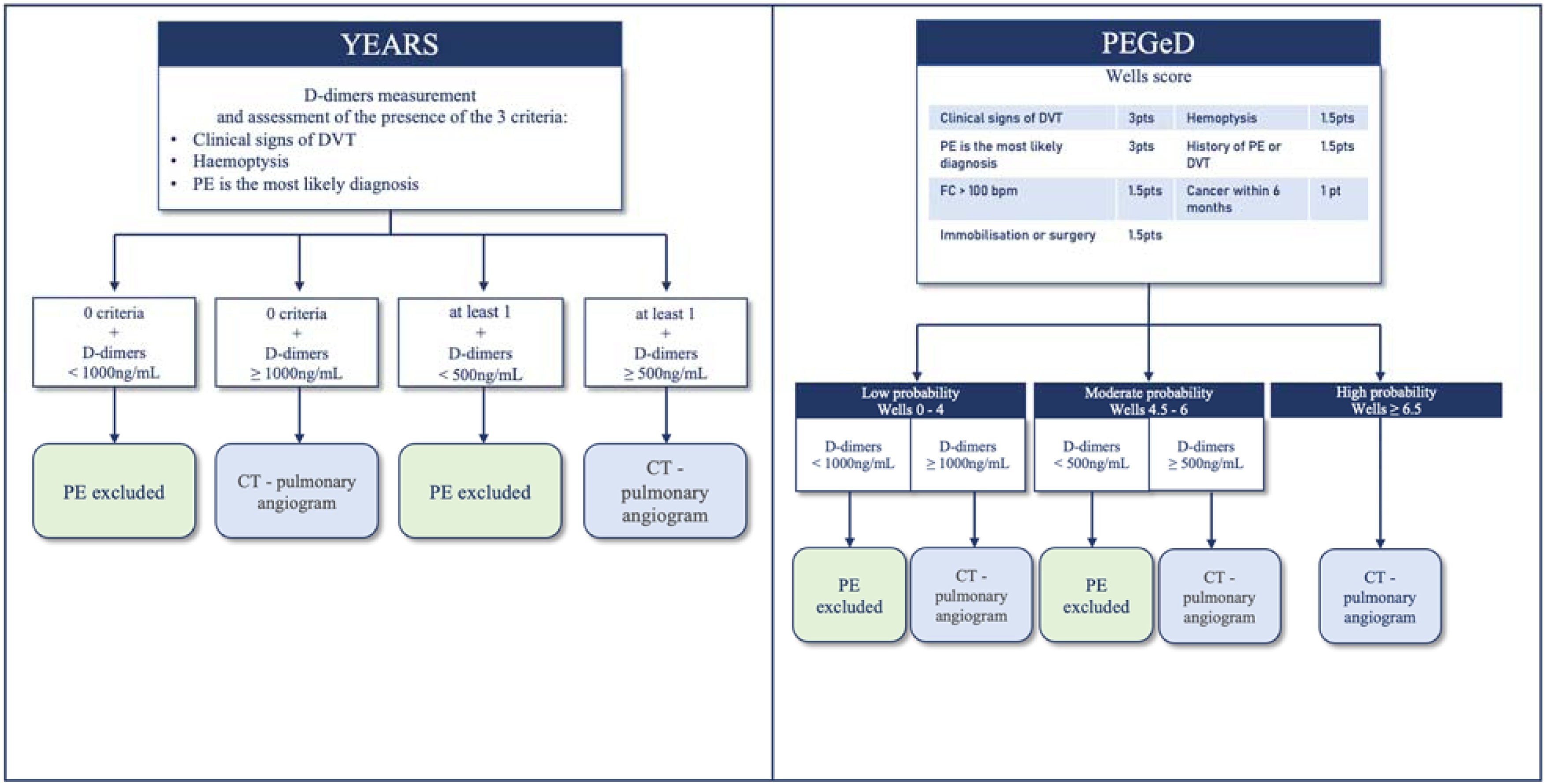
Two diagnostic strategies in dyspneic patients with suspected pulmonary embolism. Abbreviations: DVT, deep vein thrombosis; PE, pulmonary embolism; HR, heart rate.

R3.1.1. In patients with a very low clinical suspicion of pulmonary embolism (PE), the use of the Pulmonary Embolism Rule-out Criteria (PERC) is recommended to safely exclude the diagnosis without the need for further testing.
GRADE 1, High level of evidence (strong agreement)
R3.1.2. In patients with suspected pulmonary embolism (PE), the YEARS and PEGeD diagnostic strategies are probably preferable. These approaches have been shown to be safe and to reduce the number of unnecessary diagnostic tests, compared to strategies based on the revised Geneva score or the Wells score combined with age-adjusted D-dimer thresholds (Fig. 1).
GRADE 2, Moderate level of evidence (strong agreement)
R3.1.3. In patients with suspected pulmonary embolism (PE), the experts suggest that the chosen diagnostic strategy - regardless of which validated algorithm is used (e.g., PERC, YEARS, PEGeD, Geneva, or Wells) - should be followed through to completion in order to ensure the safety and reliability of the diagnostic process.
Expert opinion (strong agreement)

### Rationale

Dyspnea is the most frequent symptom among patients with pulmonary embolism (PE). However, the clinical manifestations are often nonspecific [57]. In patients with underlying heart failure or chronic pulmonary disease, worsening dyspnea may be the only presenting symptom of PE, making the diagnosis particularly challenging. Over the past decades, diagnostic strategies for PE have been associated with an increasing overuse of imaging and laboratory tests [58,59]. This overuse has led to a growing incidence of low-risk PE cases, including subsegmental emboli frequently managed in the outpatient setting, with minimal impact on overall mortality [58,59]. Consequently, these trends have raised concerns about overdiagnosis and resource consumption, prompting the development of diagnostic strategies aimed at preserving safety while reducing unnecessary investigations.

The Pulmonary Embolism Rule-out Criteria (PERC), developed and validated by Kline et al., are intended for use in patients with very low pre-test probability of PE [60]. The rule consists of eight clinical criteria. When all are negative in a patient with low clinical suspicion, the likelihood of PE is sufficiently low to obviate further testing, with a false-negative rate below 2% [60]. This rule has been validated in multiple studies, with an estimated failure rate of about 1% [[61], [62], [63]]. In Europe, two prospective studies - PERCEPIC and PROPER - reported very low false-negative rates of 1.2% (95%CI: 0.4–2.9%) and 0.1% (95%CI: 0−0.8%), respectively [64,65]. Since the pivotal study by *Righini* et al., French and European guidelines have endorsed the use of the revised Geneva or Wells scores in combination with age-adjusted D-dimer thresholds [[66], [67], [68]]. However, evidence remains contradictory regarding the use of unstructured or implicit clinical probability assessment, and no randomized controlled trial has evaluated this approach. In this context, newer diagnostic strategies have been developed and validated to improve diagnostic efficiency while maintaining patient safety.

The YEARS algorithm was designed to reduce the number of unnecessary computed tomography pulmonary angiograms (CTPAs). It incorporates three clinical items: signs of deep vein thrombosis (DVT), hemoptysis, and PE being the most likely diagnosis. In patients with none of these items, a higher D-dimer threshold of 1,000 µg/L is applied. This strategy led to a 14% reduction in CTPA use, with a failure rate of only 0.61% (95% CI: 0.4–1%) [[69], [70], [71]]. Some authors have suggested that the “hemoptysis” item could be removed without compromising diagnostic performance [72]. However, the presence of respiratory distress with hemoptysis remains an indication for thoracic CTPA, rendering probabilistic approaches less relevant in such cases. Notably, the YEARS strategy requires systematic D-dimer testing for all patients.

The PEGeD (Pulmonary Embolism Graduated D-dimer) approach applies a similar probability-adapted D-dimer threshold, using the Wells score. A D-dimer cutoff of 1,000 µg/L is applied in patients with low clinical probability, and 500 µg/L in those with moderate probability. This strategy avoided 17.6% of CTPAs compared to conventional approaches and reported a 0% failure rate (95% CI: 0−0.29%) [73]. To date, no high-level evidence has challenged the safety of the YEARS and PEGeD algorithms [74,75]. These strategies consistently remain below the predefined safety threshold established by the *Dronkers* et al. meta-analysis on behalf of the ISTH subcommittee [76].

Regardless of the diagnostic strategy chosen, it is essential to adhere to the complete diagnostic pathway through to confirmation or exclusion of PE. In a multicenter study involving 116 emergency departments in France and Belgium and including 1,529 patients, non-adherence to the recommended diagnostic strategy was independently associated with an increased risk of venous thromboembolic events within 3 months (adjusted OR = 4.29; 95%CI: 1.45–12.70) [77]. Currently, two conceptual approaches coexist (Fig. 1): one favors simplification and clinical intuition [78], while the other emphasizes precision through formalized scoring systems, potentially supported by cognitive aids - such as the 4PEPS strategy or IPD meta-analyses-based algorithms [[79], [80], [81]]. Prospective validations of these approaches are underway (e.g., NCT06190392, NCT06015529).

In pregnant women, the prevalence of confirmed PE remains relatively low, estimated between 2% and 7% [68]. D-dimer testing retains a role in excluding PE in cases of low clinical probability, although its diagnostic yield is reduced due to physiologically elevated D-dimer levels during pregnancy. The YEARS algorithm was prospectively validated in this population, showing a 3-month thromboembolic event rate of 0.2% (95%CI: 0.04–1.2%) and a 39% reduction in CTPA use (95%CI: 35–44%) [82]. Although CT pulmonary angiography remains the diagnostic gold standard, ventilation/perfusion (V/Q) scintigraphy remains a valid alternative when CTPA is contraindicated.

In patients presenting with respiratory distress, does blood gas analysis (arterial or venous), combined with peripheral oxygen saturation (SpO₂) measurement, allow accurate identification of hypoxemia with or without associated hypercapnia?

**Table:** 

R3.2.1. Venous blood gas analysis is not recommended for assessing the degree of hypoxemia
GRADE 1, High level of evidence (strong agreement)
R3.2.2. Peripheral oxygen saturation (SpO₂) alone is probably sufficient to assess the degree of oxygenation. In patients breathing ambient air, an SpO₂ >96% makes a PaO₂ <60 mmHg unlikely. In patients with chronic obstructive pulmonary disease (COPD), an SpO₂ >92% similarly makes significant hypoxemia unlikely.
GRADE 2, Moderate level of evidence (strong agreement)
R3.2.3. The experts suggest that venous blood gas analysis may be used to assess carbon dioxide levels. A normal venous PvCO₂ (<45 mmHg) makes a PaCO₂>50 mmHg unlikely.
Expert opinion (strong agreement)
R3.2.4. Arterial blood gas analysis is probably the reference standard, particularly in complex clinical situations
GRADE 2, Moderate level of evidence (strong agreement)

### Rationale

As expected from basic physiology, venous and arterial blood gas measurements yield discordant results for oxygenation parameters, and venous values cannot be reliably used to assess the severity of hypoxemia [83,84]. In contrast, multiple studies have shown a strong correlation between peripheral oxygen saturation (SpO₂) and arterial oxygen saturation (SaO₂) [[85], [86], [87], [88]]. In patients with acute exacerbations of chronic obstructive pulmonary disease (COPD), a multicenter study by *Garcia-Gutierrez* et al. involving 2,181 patients across 16 hospitals found an excellent correlation between ambient-air SpO₂ and PaO₂ (AUC 0.97; 95% CI [0.96−0.98]), identifying a SaO₂<90% as a strong predictor of PaO₂ <60 mmHg [89]. Similarly, *Kelly* et al. reported that an SpO₂ <92% yielded a sensitivity of 100% and specificity of 86% for detecting a PaO₂ <60 mmHg [90]. To our knowledge, the only study that proposes using the combined interpretation of venous blood gases and SpO₂ in the management of patients with respiratory distress and/or those with suspected acute respiratory failure is the prospective, controlled monocenter study by *Golub* et al. (n = 102) [91]. However, this approach has significant limitations, notably the absence of associated shock and the requirement for low FiO₂. Regarding carbon dioxide (CO₂) assessment, several reviews and meta-analyses have documented substantial differences between arterial and venous PCO₂ measurements - sometimes as high as 20 mmHg - rendering venous samples unreliable for evaluating hypercapnia in acute settings [92,94]. Some authors have suggested using a correction factor (e.g., subtracting 5.8–6.8 mmHg from PvCO₂), but this has not been validated in randomized prospective trials [84,89,90]. Nevertheless, other authors have shown that a normal PvCO₂ has good predictive value for excluding elevated PaCO₂ [90,93]. A review by Bloom et al., including 12 studies and 852 patients, concluded that a normal venous PCO₂ was a reliable indicator for ruling out hypercapnic respiratory distress [95]. For CO₂ assessment, pH measurement is also crucial. Venous pH has been validated as an acceptable surrogate for arterial pH, with a meta-analysis by *Byrne* et al. (15 studies, 1,747 patients) reporting a mean difference of +0.033 (95% CI [0.029−0.038]) between venous and arterial values [96]. Finally, in critically ill or diagnostically complex cases, arterial blood gas analysis remains a cornerstone of the diagnostic work-up for patients with respiratory distress, regardless of its underlying etiology [1].

In patients presenting with respiratory distress, does the combination of venous and arterial blood gas analysis with peripheral oxygen saturation (SpO₂) improve clinical decision-making (treatment, patient disposition, monitoring) compared to SpO₂ alone?

**Table:** 

R3.3.1. No recommendation can be issued regarding the use of venous blood gas analysis in addition to peripheral oxygen saturation (SpO₂) measurement to improve clinical decision-making
No recommendation
R3.3.2. The experts suggest that arterial blood gas analysis should not be performed systematically to guide clinical decision-making (treatment, patient disposition) or monitoring in patients presenting with respiratory distress with or without signs of severity. Arterial blood gas analysis may be considered in the presence of respiratory distress to confirm the need for ventilatory support or to guide patient disposition, particularly in cases where SpO₂ is unreliable or cannot be measured, or to confirm and quantify hypercapnia and respiratory acidosis when venous PCO₂ is elevated.
Expert opinion (strong agreement)

### Rationale

To date, no studies have evaluated whether venous blood gas analysis improves clinical decision-making in terms of treatment, disposition, or monitoring in patients with respiratory distress. To our knowledge, no evidence exists supporting the combined use of venous blood gases (adjusted or not) and pulse oximetry (SpO₂) to guide therapeutic decisions or ICU referral. Future studies are needed to determine whether the combination of venous blood gas analysis and pulse oximetry could help clinicians assess the severity of acute respiratory failure, identify the need for ventilatory support, or determine appropriate disposition to a higher level of care.

No studies have directly compared arterial blood gas analysis to SpO₂ alone for guiding therapeutic decisions or ICU admission in patients with respiratory distress of any etiology. However, arterial blood gas parameters are commonly used in clinical practice to support decisions regarding the initiation of non-invasive ventilation (NIV), invasive mechanical ventilation, or ICU referral. Most clinical trials assessing the indications and outcomes of non-invasive ventilatory support have included patients on the basis of their arterial blood gas values. Early arterial blood gas sampling allows the detection of moderate hypercapnic respiratory acidosis or severe hypoxemia. In hypercapnic patients, there is strong evidence supporting the use of bilevel non-invasive ventilation in those with arterial pH < 7.35 (in the absence of a metabolic cause for the acidosis) [[97], [98], [99], [100]]. Recent guidelines do not recommend the use of NIV in patients without moderate to severe acidosis, as it is often poorly tolerated and not associated with improved outcomes [97,101].

In hypoxemic patients, the decision to initiate non-invasive oxygenation strategies (e.g., high-flow nasal oxygen or NIV) is not always based on arterial blood gases alone. Several studies have used SpO₂ values combined with clinical parameters upon admission [[102], [103], [104], [105], [106], [107]]. However, the prognostic effects of high-flow nasal oxygen (HFNO) in de novo acute respiratory failure were demonstrated in patients selected on the basis of arterial blood gas values, particularly a PaO₂/FiO₂ ratio<300 associated with a respiratory rate >25 breaths/min [108]. Similarly, non-invasive ventilation has been proposed as an alternative to invasive ventilation in acute hypoxemic respiratory failure with a PaO₂/FiO₂ ratio ≤200, although prognostic benefits remain uncertain. In hypercapnic patients, failure of NIV and the need for intubation have been associated with lack of improvement in arterial pH at 1 hour [109], or a pH ≤ 7.25 at admission [110,111]. Recent studies have proposed clinical scores to predict NIV failure, such as the HACOR score (Heart rate, Acidosis, Consciousness, Oxygenation, Respiratory rate) and the NIVO score, both of which require arterial pH measurement [112,113].

For hypoxemic patients, the ROX index, proposed by *Roca et al.,* is a non-invasive tool used to predict the risk of HFNO failure in acute hypoxemic respiratory failure [114,115]. It is calculated as (SpO₂/FiO₂) / respiratory rate, and does not require arterial blood gas analysis. Repeated measurements of the ROX index, with thresholds ≥4.88 (low risk) and <2.85 (high risk) for intubation, can help guide treatment decisions [115]. The ROX index has also been evaluated for predicting intubation risk in patients treated with NIV, with comparable performance to its use in HFNO-treated patients, and similar to PaO₂/FiO₂-based assessments [116]. In contrast, the HACOR score, developed to predict NIV failure in acute respiratory failure, and its updated version, require arterial blood gas measurements and have shown superior predictive performance compared to non-invasive indices [117].

In patients presenting to the Emergency Department (ED) with acute dyspnea with or without associated respiratory and suspected lower respiratory tract infection, should C-reactive protein (CRP) or procalcitonin (PCT) be measured to support the diagnosis of bacterial respiratory infection and guide initial therapeutic management?

**Table:** 

R3.4.1 - There is insufficient evidence to recommend the measurement of C-reactive protein (CRP) to guide the initiation of antibiotic therapy in patients presenting to the ED with acute dyspnea with or without associated respiratory distress and suspected lower respiratory tract infection
No recommendation
R3.4.2 - There is insufficient evidence to recommend regarding the use of procalcitonin (PCT) to guide the initiation of antibiotic therapy in patients presenting to the ED with acute dyspnea with or without associated respiratory distress and suspected lower respiratory tract infection.
No recommendation

### Rationale

The utility of C-reactive protein (CRP) measurement to reduce antibiotic exposure in adults with suspected lower respiratory tract infection and respiratory distress has been explored primarily in out-of-hospital settings. Several randomized controlled trials have investigated point-of-care CRP testing in ambulatory adults [[118], [119], [120], [121]], in patients with COPD [122], and in institutionalized elderly patients [123], often as part of a CRP-guided antibiotic initiation strategy, either alone or in combination with physician education programs [118,120]. With the exception of two trials [119,121], these studies generally demonstrated a higher rate of antibiotic withholding in the CRP-guided arms. However, none of these studies were conducted in emergency departments or among hospitalized patients, and therefore no recommendation can currently be made for such populations.

Several randomized controlled trials have assessed the role of procalcitonin (PCT) in reducing antibiotic use in adults with suspected bacterial lower respiratory tract infections and acute dyspnea, either in the ED or in hospitalized settings [[124], [125], [126], [127]]. Included patient populations varied across studies, and commonly included those with community-acquired pneumonia (CAP), acute exacerbation of COPD, acute bronchitis, or asthma exacerbation. Notably, the proportion of patients with confirmed pneumonia ranged from 19% [124] to 100% [125]. These five trials (total n = 2,364) used a PCT threshold of 0.25 ng/mL, either as a single measurement to guide antibiotic initiation [127,128], or as part of repeated assessments during hospitalization to guide antibiotic discontinuation [[124], [125], [126]]. Primary endpoints were generally related to antibiotic exposure [124,125,127,128], or were composite outcomes involving prognosis, side effects, and duration of antibiotic treatment [126]. Overall, with the exception of one study [124], these trials reported significant reductions in antibiotic exposure (ranging from 15% to 50%) without impacting patient prognosis. For instance, in the 2004 study by Christ-Crain et al., antibiotics were withheld in 66% of patients in the PCT-guided arm compared to only 17% in the control arm (p < 0.0001), with no difference in clinical outcomes [128]. An international observational study also showed that the reduction in antibiotic use was most pronounced when clinicians adhered closely to the PCT algorithm [129]. Similar findings have been observed in ambulatory care settings [[130], [131], [132]], and in more narrowly defined populations such as those with asthma exacerbation [133] or acute COPD exacerbation [134]. These results have been supported by multiple meta-analyses [[135], [136], [137]]. However, the data remain heterogeneous and should be interpreted with caution. For example, a large negative RCT involving over 1,600 patients with suspected lower respiratory tract infection failed to show benefit, as did a French multicenter RCT evaluating PCT-guided therapy in patients with pneumonia [138,139]. In patients over 80 years of age, a single clinical trial explored PCT use only for antibiotic discontinuation during hospitalization [140]. In more severely ill populations requiring ICU admission, two trials suggested that PCT may help shorten antibiotic duration in cases of sepsis of presumed pulmonary origin [141,142]. However, the only ICU trial that tested PCT-guided antibiotic withholding at admission (in patients with acute COPD exacerbation) showed concerning results: among those in whom antibiotics were withheld, PCT-guided management was associated with increased mortality (31% vs. 12%, p = 0.015) [143].

In patients presenting with acute dyspnea with or without associated respiratory distress and diagnostic uncertainty, what is the value of measuring BNP (or NT-proBNP) for the diagnosis of acute heart failure (AHF)?

**Table:** 

R3.5.1. Measurement of natriuretic peptides is probably recommended to help rule out the diagnosis of acute heart failure (AHF), given their excellent negative predictive value across various clinical settings, except in obese patients. This measurement should only be performed and interpreted in conjunction with clinical information.
GRADE 2, Moderate level of evidence (strong agreement)

### Rationale

The etiological diagnosis of respiratory distress in emergency settings is particularly challenging. This difficulty arises from the non-specific nature of symptoms, the frequent presence of comorbidities, and the limited diagnostic performance of routine investigations such as arterial blood gas analysis, electrocardiogram (ECG), and chest radiography - excluding, in this context, lung and pleural ultrasound [144]. However, early identification of the underlying cause of dyspnea and rapid initiation of appropriate treatment are associated with improved prognosis, particularly in cases of cardiogenic pulmonary edema (CPE) [145,146]. Within this framework, the use of biomarkers to confirm or rule out CPE is a promising strategy. B-type natriuretic peptides (BNP) and their N-terminal fragment (NT-proBNP) are secreted by ventricular myocytes in response to predominantly mechanical stimuli, such as left ventricular wall stretch caused by volume overload or increased intracavitary pressure. These peptides represent quantitative biomarkers of cardiac hemodynamic stress [147]. Their clinical use, however, must be carefully contextualized. Interpretation should be integrated with clinical probability assessment, which itself is based on history-taking, physical examination, and chest X-ray findings. Furthermore, BNP and NT-proBNP values should be interpreted in light of renal function and body mass index (BMI), which are the two main confounders affecting plasma concentrations. Lastly, it is essential to recognize that BNP and NT-proBNP assays - whether performed as point-of-care tests or through centralized laboratory platforms - are neither interchangeable nor directly comparable [[148], [149], [150]]. In the early 2000s, multiple studies investigated the utility of BNP and NT-proBNP as diagnostic tools for acute heart failure in the ED [151,152]. The sensitivity and negative predictive value (NPV) of BNP vary according to the cutoff used and the underlying prevalence of heart failure in the population studied. Nevertheless, most authors have proposed relatively consistent thresholds, typically BNP levels below 100 pg/mL or NT-proBNP levels below 300 to 500 pg/mL. Below these cutoffs, the NPV for excluding acute heart failure is close to 90%, making natriuretic peptide testing a valuable tool for ruling out the diagnosis in patients with diagnostic uncertainty.

In patients presenting with acute dyspnea with or without associated respiratory distress, what diagnostic changes are induced by the use of cardiac and lung ultrasound compared to a strategy without ultrasound?

**Table:** 

R3.6.1. The use of integrated cardiac and lung ultrasound is probably recommended to assist in determining the underlying cause of acute dyspnea.
GRADE 2, Moderate level of evidence (strong agreement)

### Rationale

Lung ultrasound has demonstrated superior diagnostic efficiency compared to chest X-ray for the evaluation of cardiogenic pulmonary edema, pneumonia, and pleural effusions - both liquid and air-related. This technique is rapid, non-irradiating, performed at the bedside by the treating physician, and provides immediate results. Its performance has been particularly well documented in the diagnosis of community-acquired pneumonia, with a meta-analysis reporting an area under the ROC curve (AUC) of 0.93 [153]. Nevertheless, its sensitivity may be limited in cases of centrally located lung infections without peripheral involvement. Similarly, in cases of acute left heart failure, the presence of a bilateral B-line pattern is strongly associated with pulmonary edema, with an odds ratio (OR) of 7.4 (95% CI: 4.2–12.8), while its absence argues against the diagnosis (OR = 0.16; 95% CI: 0.05−0.51) [144]. In a multicenter prospective study of 1,005 patients, the use of lung ultrasound changed the presumed etiology (cardiac vs. non-cardiac) in 19% of cases compared to standard diagnostic approaches [154]. When thoracic ultrasound includes both lung and cardiac assessment, diagnostic performance is further improved. A prospective randomized study comparing standard diagnostic workup with a protocol including thoracic ultrasound found a 24% improvement (95% CI: 15–33) in diagnostic accuracy relative to a predefined reference diagnosis [155]. Other non-randomized studies have also reported enhanced diagnostic performance with integrated thoracic ultrasound [[156], [157], [158]]. In contrast, the diagnostic accuracy of cardio-pulmonary ultrasound for pulmonary embolism (PE) is limited. In non-shocked patients, its sensitivity is insufficient to confirm or exclude PE [159,160]. However, in the context of circulatory shock, direct visualization of thrombi in the right heart chambers confirms the diagnosis of massive pulmonary embolism [161]. Additionally, in hemodynamically unstable patients with suspected PE, echocardiographic signs of acute cor pulmonale - such as right ventricular dilatation and paradoxical septal motion - are considered diagnostic [162].

Ultrasound is also superior to bedside chest X-ray for the diagnosis of pneumothorax, offering faster assessment and greater sensitivity, especially in small-volume pneumothoraxes. Diagnosis relies on the assessment of four dynamic sonographic signs: pleural sliding, presence of B-lines, the lung point, and the lung pulse. The presence of pleural sliding and/or B-lines rules out pneumothorax, although their absence is not specific for its presence. Conversely, the presence of a lung point is 100% specific for pneumothorax, but its sensitivity is only around 60%. The diagnostic value of the lung pulse has not been robustly established in prospective studies. Although highly specific, the echographic diagnosis of pneumothorax is also operator-dependent [163]. In patients presenting with respiratory distress in a suggestive clinical context, lung ultrasound enables rapid exclusion of moderate to large pneumothorax and may allow early diagnosis. In patients with respiratory distress and suspected chronic obstructive pulmonary disease (COPD) exacerbation, ultrasound contributes diagnostically by helping to rule out concurrent conditions such as pulmonary edema, parenchymal consolidation, or pleural effusion - whether gaseous or liquid [163].

In patients presenting with respiratory distress and suspected community-acquired pneumonia (CAP), what diagnostic changes are induced by low-dose chest CT compared to a standard diagnostic strategy?

**Table:** 

R3.7.1. The experts suggest performing a low-dose chest CT scan in cases of diagnostic uncertainty regarding suspected community-acquired pneumonia, or as a first-line imaging modality when the standard strategy does not provide sufficiently high-quality images.
Expert opinion (strong agreement)

### Rationale

The most commonly used imaging modality in the initial evaluation of suspected community-acquired pneumonia (CAP) remains the frontal chest radiograph, which offers acceptable diagnostic performance, with a reported sensitivity of 71% and specificity of 86%. However, image quality and diagnostic yield can be significantly impaired in the emergency department setting due to suboptimal acquisition conditions. Low-dose chest computed tomography (CT) is an imaging technique that provides high-resolution pulmonary parenchymal visualization sufficient to support a diagnostic decision. Its diagnostic performance is nearly equivalent to that of standard-dose chest CT, with similar costs and procedural constraints [164]. Its primary advantage lies in significantly lower radiation exposure, making it particularly attractive in populations where radiation protection is critical, such as younger patients or women of childbearing age [165]. Nevertheless, low-dose CT cannot be reliably performed in patients with a body mass index (BMI) greater than 35 kg/m² [166].

Although it is technically feasible on the majority of CT scanners currently available in France, access to low-dose CT remains limited, making it impractical to propose its systematic use in all patients with suspected CAP. Local expertise, equipment availability, and institutional workflows must also be taken into account. At present, there are no formal recommendations - outside the context of COVID-19 - that support the routine use of CT as a first-line diagnostic tool in the evaluation of suspected CAP in emergency departments. Most existing guidelines are, however, relatively outdated (Table 1).

**Table 1:** Summary of current guidelines regarding imaging strategies.

Country, Year	Ref.	Content
United States, 2007	[167]	CT is described as more sensitive, but its specificity is considered limited.
United States, 2019	[168]	The guideline does not address diagnostic CT; it considers CAP diagnosis to rely on the clinical and radiographic combination.
Netherlands, 2018	[169]	The guideline highlights the superior diagnostic performance of CT and its broader use due to ED availability, but concludes that current evidence does not support first-line use.
Switzerland, 2004	[170]	Thoracic CT is not mentioned in this guideline.
UK (BTS), 2004	[171]	CT may be useful in patients with diagnostic uncertainty.
UK (NICE), 2014	[172]	Thoracic CT is not mentioned in this guideline.

Abbreviations: CAP: community-acquired pneumonia; CT: computed tomography; ED: Emergency Department.

Several recent clinical studies have evaluated the diagnostic performance of CT (particularly low-dose CT) in patients with suspected CAP. In one study, routine CT scanning in patients presenting to the emergency department for suspected CAP led to reclassification of the diagnosis in approximately one-third of cases, regardless of the findings on chest X-ray [173]. Two additional studies demonstrated that early CT imaging, performed within 4 hours in the ED or within 72 hours in geriatric settings, improved diagnostic accuracy based on clinical and radiological criteria in 59% and 45% of cases, respectively [174,175]. Another study showed that CT imaging improved the quality of the final diagnosis when performed during the ED stay [176]. However, one study tempered these findings by reporting that CT altered the diagnosis in only 9% of cases compared to standard chest radiography [177].

Low-dose CT may therefore provide greater diagnostic precision in selected patients, particularly in situations of diagnostic uncertainty or when lower lobe involvement is suspected [177,184]. Its use in evaluating non-traumatic thoracic conditions in emergency settings has been associated with an improved final diagnostic rate, especially in patients with atypical clinical presentations [178,179]. Nonetheless, data on morbidity, mortality, and healthcare utilization remain limited, and the overall clinical impact of low-dose CT is still uncertain. Some studies have suggested that it may influence clinical decisions, particularly regarding antibiotic initiation, leading to prescriptions more aligned with current guidelines [180,183]. While it does not appear to reduce the total number of antibiotic days, CT imaging has been associated with significantly shorter emergency department stays and reduced need for further imaging during hospitalization [179]. A retrospective study found that CT reduced the time to clinical decision-making in patients admitted for respiratory distress (6.9 vs. 9.5 hours) [181]. Other studies suggest it may reduce hospital length of stay by approximately two days [176,177], although this has not been consistently confirmed. There appears to be no significant impact on ICU admission, mechanical ventilation, development of septic shock, or in-hospital mortality. To date, no conclusive studies have evaluated the cost-effectiveness of low-dose CT in this context, except in COVID-19, where CT-based strategies have been shown to be cost-efficient [182].

In patients admitted to the Emergency Department (ED) with respiratory distress, what age-specific prognostic criteria are essential for predicting in-hospital survival in older adults compared to younger patients, excluding any consideration of treatment limitation decisions?

**Table:** 

R3.8.1. The experts suggest assessing functional dependency and frailty in elderly patients presenting with respiratory distress to the ED in order to predict in-hospital survival. Beyond chronological age, there are no specific prognostic criteria identified for in-hospital mortality.
Expert opinion (strong agreement)

### Rationale

There are various approaches to defining "older adults" depending on the context and objectives of analysis. The World Health Organization (WHO) considers individuals over the age of 60 as older persons, whereas most epidemiological studies and clinical trials conducted in high-income countries (e.g., USA, UK, Europe) typically use a threshold of 65 years. The term "very old" generally refers to individuals aged 85 years or older. In this guideline, we did not adopt a fixed age cutoff for defining "older" or "elderly" patients, as demographic transitions in Western countries are rapidly rendering prior definitions obsolete. Consequently, the definition of an "older patient" varies considerably across the literature, making direct comparison between studies difficult.

The association between age and increased mortality is well established in many clinical scenarios. Age has consistently been shown to be an independent risk factor for in-hospital mortality, even after adjusting for other variables [[183], [184], [185]]. However, only a few descriptive or observational studies have specifically investigated age-related prognostic factors in elderly patients presenting with respiratory distress to the Emergency Department. Importantly, no prognostic factor uniquely associated with age has been identified as predictive of in-hospital mortality in this population. In studies with a low level of evidence, prognostic factors observed in older adults were largely similar to those found in younger populations and included underlying comorbidities such as chronic kidney disease, chronic obstructive pulmonary disease (COPD), and heart failure, as well as the severity of the index condition [[186], [187], [188]]. Other conditions common in the aging population - such as neurocognitive disorders, frailty, malnutrition, and falls - have been independently studied in observational cohorts. While these conditions increase in prevalence with age, they are not exclusively age-related and thus cannot be considered specific to elderly individuals. Among older adults with respiratory distress, the most frequently studied prognostic factor is loss of functional independence, typically assessed using autonomy scales such as the Katz Activities of Daily Living (ADL) scale or the Barthel Index [189,190]. Confusion, neurocognitive disorders, and frailty have also been evaluated, although the number of available studies remains low, with small sample sizes and consequently limited levels of evidence [[191], [192], [193]]. Each of these factors has been associated with increased in-hospital or 30-day mortality in patients aged 65 years or older.

In critically ill older patients, the Clinical Frailty Scale (CFS) has been increasingly studied as a tool for predicting mortality. Frailty is defined as “a clinical state in which there is an increased vulnerability to developing dependency and/or mortality when exposed to a stressor” [194]. A robust association has been demonstrated between the CFS and both short- and long-term mortality in patients over 80 years of age admitted to intensive care units, with acute respiratory failure being a common reason for admission [[195], [196], [197], [198]]. The CFS appears to predict prognosis independently of any decisions regarding therapeutic limitation and was not significantly improved by the addition of other geriatric assessment tools such as the ADL scale, the IQCODE (Informant Questionnaire on Cognitive Decline in the Elderly), or the Comorbidity and Polypharmacy Score [199]. Although widely used as a prognostic tool in older populations, the CFS is not specific to any fixed age group. Its association with in-hospital mortality has also been reported in younger ICU patients, including those with acute respiratory failure (14%) or sepsis (23%) [200].

Recommandation Formalisée d’Experts: 50% SFMU - 50% SRLF, SFMU leader.

Co-Organisateurs: Pierrick Le Borgne (Strasbourg), Arnaud W. Thille (Poitiers).

Co-Coordonnateurs d'experts: Philippe Leconte (Nantes), Nicolas Terzi (Rennes)

This document was endorsed by the Guidelines Committee of the French Society for Emergency Medicine (SFMU) in March 2025 and by its Executive Committee in May 2025, as well as by the Guidelines Committee of the French Intensive Care Society (SRLF) in March 2025 and its Executive Committee in May 2025.

## Reviewers

Guidelines Committee of the French Society for Emergency Medicine: J. Guenezan (Poitiers), D. Douillet (Angers), J.-B. Bouillon (Clermont-Ferrand), P. Catoire (Paris), R. Chocron (Paris), X. Dubucs (Toulouse), Charles Grégoire (Bruxelles), Amélie Vromant (Paris), M. Jonchier (La Rochelle), P. Le Borgne (Strasbourg), N. Peschanski (Rennes), G. Rousseau (Tours), N. Tiberti (Aix-en-Provence), Y.-E. Claessens (Monaco), R. Kadji-Kalabang (Melun), T. Markarian (Marseille).

Executive Committee of the French Society for Emergency Medicine: S. Charpentier (Présidente, Toulouse), D. Savary (Angers), C. Pradeau (Bordeaux), P. Jabre (Paris), J.-P. Fontaine (Paris), A. Chauvin (Paris), T. Chouhied (Nancy), F. Dumas (Paris), O. Mimoz (Poitiers), J. Contenti (Nice), P. Ray (Dijon), N. Termoz-Masson (Grenoble), Y. Yordanov (Paris), X. Bobbia (Montpellier).

Guidelines Committee of the French Intensive Care Society: AW. Thille (Secrétaire, Poitiers), Julie Starck (Paris), I. Adda (Le Kremlin-Bicêtre), N. Aissaoui (Paris), J. Blauhorn (Strasbourg), PF. Dequin (Tours), S. Hraiech (Marseille), LM. Jandaux (Strasbourg), M. Jezequel (Paris), M. Marzouk (Arras), JY. Mootien (Mulhouse), M. Simon (Lyon).

Executive Committee of the French Intensive Care Society: M. Fartoukh (Présidente, Paris), S. Nseir (Lille), K. Abidi (Rabat, Maroc), AS. Debue (Paris), J. Helms (Strasbourg), G. Carteaux (Créteil), B. Gaillard-Leroux (Nantes), JP. Frat (Poitiers), JP. Quenot (Dijon), S. Leteurtre (Lille), J. Hayon (Poissy), W. Bougouin (Massy), N. Perinel (Lyon), N. Bige (Villejuif), A. Laurent (Dijon), G. Maerckx (Bruxelles, Belgique), F. Pene (Paris), C. Petyt (Paris).

## CRediT authorship contribution statement

Dr. Pierrick Le Borgne had full access to all of the data in the study and takes responsibility for the integrity of the data and the accuracy of the data analysis. All authors contributed to drafting of the work, revising it critically for important intellectual content and approved the final version of the manuscript. All authors give their agreement to be accountable for all aspects of the work, and ensure the accuracy and integrity of any part of the work.

## Consent for publication

Not applicable.

## Ethics approval and consent to participate

Not applicable

## Funding

None.

## Availability of data and material

Not applicable

## Declaration of competing interest

Arnaud W. Thille reports having received research funding as well as personal fees (including honoraria for lectures and reimbursement of travel and accommodation expenses for participation in scientific conferences) from Fisher & Paykel.

Adrien Basset declares a conflict of interest with Fisher & Paykel.

Anne Sophie Boureau declare aucun conflit d’intérêt en lien avec cette RFE. Autres conflits intérêts: Pfizer and Astrazeneca.

Yann-Erick Claessens declare des COI with Roche Diagnostics, bioMérieux, Beckman Coulter, Thermo Fisher, Siemens, ViroGates, Sanofi, Air Liquid and Abbott.

Jean-Luc Diehl declare des COI with GE Medical systems, Fresenius Medical Care and Baxter

Jérémy Guenezan: Abbott, Siemens, Biomerieux, Viatris, Becton Dickinson, Roche

Pierre Hausfater has received reading and consultancy honoraria from Beckman Coulter, bioMérieux, and Siemens; he has also received clinical study funding from Beckman Coulter, bioMérieux, and Sysmex.

Erwan L'HER is co-founder and shareholder of Oxynov Inc. - Canada and Ivanaë Médical - France. He is consultant for GE Healthcare and Sedana Medical

Nicolas Marjanovic has participated in symposia sponsored by Fisher & Paykel Healthcare and Aerogen, and has received research funding from Aerogen.

Paul-Georges Reuter: pas de COI

Guillaume Voiriot has received research funding from bioMérieux and SOS Oxygène, as well as support for congress attendance from Oxyvie.

Nicolas Terzi has received honoraria for lectures from Fisher & Paykel (outside the scope of this work), as well as congress attendance support from Pfizer (travel expenses) and non-financial support from Gilead (also outside the scope of this work).

Philippe Leconte serves as a speaker for GE Healthcare.

All other authors declare no conflicts of interest.
